# Differential expression of a prophage-encoded glycocin and its immunity protein suggests a mutualistic strategy of a phage and its host

**DOI:** 10.1038/s41598-019-39169-3

**Published:** 2019-02-26

**Authors:** Emma L. Denham, Sjouke Piersma, Marleen Rinket, Ewoud Reilman, Marcus C. de Goffau, Jan Maarten van Dijl

**Affiliations:** 1 University of Groningen, University Medical Center Groningen, Department of Medical Microbiology, Hanzeplein 1, P.O. Box 30001, 9700 RB Groningen, The Netherlands; 2 Present Address: Department of Biology and Biochemistry, University of Bath, Bath, UK; 3 Present Address: Wellcome Sanger Institute, Cambridge, UK

## Abstract

Sublancin 168 is a highly potent and stable antimicrobial peptide secreted by the Gram-positive bacterium *Bacillus subtilis*. Production of sublancin gives *B*. *subtilis* a major competitive growth advantage over a range of other bacteria thriving in the same ecological niches, the soil and plant rhizosphere. *B*. *subtilis* protects itself against sublancin by producing the cognate immunity protein SunI. Previous studies have shown that both the *sunA* gene for sublancin and the *sunI* immunity gene are encoded by the prophage SPβ. The *sunA* gene is under control of several transcriptional regulators. Here we describe the mechanisms by which *sunA* is heterogeneously expressed within a population, while the *sunI* gene encoding the immunity protein is homogeneously expressed. The key determinants in heterogeneous *sunA* expression are the transcriptional regulators Spo0A, AbrB and Rok. Interestingly, these regulators have only a minor influence on *sunI* expression and they have no effect on the homogeneous expression of *sunI* within a population of growing cells. Altogether, our findings imply that the homogeneous expression of *sunI* allows even cells that are not producing sublancin to protect themselves at all times from the active sublancin produced at high levels by their isogenic neighbors. This suggests a mutualistic evolutionary strategy entertained by the SPβ prophage and its *Bacillus* host, ensuring both stable prophage maintenance and a maximal competitive advantage for the host at minimal costs.

## Introduction

The fitness of bacterial cells and populations belonging to a particular species is critically dependent on their ability to compete with other organisms. A highly successful competitor is the Gram-positive bacterium *Bacillus subtilis*, which thrives in the soil and plant rhizosphere. As shown through recent systems biological analyses, *B*. *subtilis* has an intricate regulatory architecture that allows it to rapidly and effectively adapt to changing conditions^1,2^. This organism has also mastered the art of adapting to a wide range of environmental stresses and insults^3,4^ and, on top of that, *B*. *subtilis* secretes a cocktail of antimicrobial compounds into its environment that is deadly for a wide range of other microbes^5,6^. These traits ensure that *B*. *subtilis* can optimally benefit from any nutrients that become available in its environment^1,2^.

**Figure 1:**
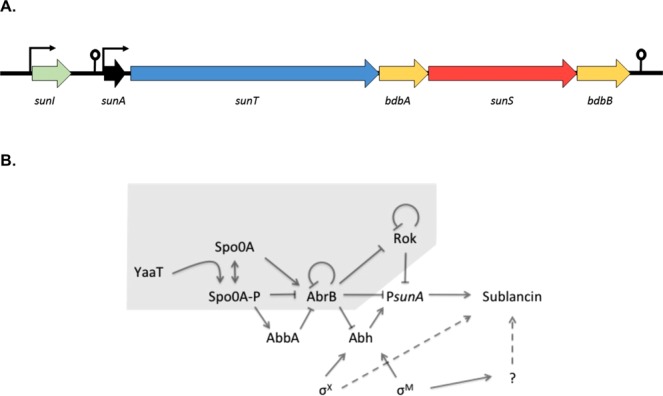
The *sunA* locus and its regulatory network. (**A**) Schematic representation of the *sunA* locus. Genes are indicated by large arrows, promoters by elbow arrows, and terminators by pins. (**B**) The regulatory network determining the expression of the *sunA* gene for sublancin as adapted from^21^. Arrows represent interactions that stimulate *sunA* expression and blunt-ended lines represent inhibitory interactions. The part of the network that determines *sunA* expression heterogeneity as determined in the present studies is indicated by grey shading.

A very potent and stable antimicrobial agent produced by *B*. *subtilis* is sublancin 168 (in short sublancin). Studies have shown that sublancin is a 37-residue peptide composed of two helices, which are connected by two disulfide bonds and an S-glucosidic linkage to a Cys residue helix-connecting loop^7–10^. As such, sublancin belongs to the family of glycocins, which share the sugar modification and require a similar machinery for biosynthesis, post-translational modification and secretion^11,12^. The glucopeptide sublancin displays bactericidal activity against a range of other Gram-positive organisms, including various bacilli. In addition, it was recently shown that sublancin also displays immunomodulatory activities^13,14^. The mechanism by which sublancin excerts its bactericidal effect is not fully understood, but it has been shown to require key factors of the sugar phophotransferase system^15^. The genes encoding for the production of sublancin are not native to the 168 strain, but they have been introduced into the genome by the SPβ phage^16–18^. The precursor to sublancin is encoded by *sunA*, which is the first gene of an operon that includes four other genes named *sunT*, *bdbA*, *sunS* and *bdbB*^7,17,18^. SunT is responsible for proteolytic removal of the leader peptide from pre-sublancin and transport of mature sublancin to the extracellular milieu^7^. Additionally, the thiol-disulfide oxidoreductase BdbB is involved in sublancin maturation by formation of the two disulfide bonds^7,19^. SunS was shown to be responsible for addition of an S-linked UDP-glucose to Cys22 of sublancin^8^. BdbA is a thiol-disulfide oxidoreductase but, unlike BdbB, BdbA is dispensable for production of active sublancin^7^. The final gene involved in the production of sublancin is *sunI*, which encodes the immunity protein that must be expressed to protect *B*. *subtilis* from the toxic effect of sublancin. Therefore, strains of *B*. *subtilis* are sensitive to sublancin if they do not produce SunI^20^. Notably, the *sunI* gene is not part of the *sunA* operon, but it is expressed from its own promoter and a rho-independent terminator is located between the two genes^2^ (Fig. 1A). Although the *sunA*, *sunT*, *bdbA*, *sunS* and *bdbB* genes are conserved in other glycocin-producing bacteria, the structures of the respective operons differ^11^.

Sublancin production is influenced by several regulatory factors as schematically represented in Fig. 1B. The two extracytoplasmic function (ECF) sigma factors σ^M^ and σ^X^ positively effect sublancin expression through the transcription factor Abh^21^. A paralogue of Abh is the transcription factor AbrB that negatively regulates *sunA* expression^22^. A further negative regulator of sublancin is Rok^23^, which is known to bind to regions of foreign DNA that have higher levels of A + T than the native DNA^24^. As well as being regulated by these ‘standard’ transcriptional regulators, the *sunA* gene can also be regulated in a condition-dependent manner. For example, the two-component regulatory system YvrGHb has been described as positively affecting the expression of the *sun* operon, although this may be through its regulation of *sigX*^25^. Furthermore, *sunA* is negatively regulated by Spx during disulfide stress^26,27^. The regulator responsible for carbon catabolite repression CcpA negatively regulates *sunA* in the presence of glucose^28^, and *sunA* also appears to be under the influence of the regulator of genetic competence and quorum sensing ComA^29^. Lastly, it has been reported that *sunA* is expressed heterogeneously^30^. This complex control of *sunA* transcription is only partly understood, and a challenge lies in dissecting the different factors responsible for this gene’s expression pattern^21^.

This study is aimed at identifying factors responsible for the heterogeneous expression of *sunA*. In addition, we assessed whether the heterogeneity in the expression of *sunA* is also reflected in the expression of the immunity gene *sunI*. For this purpose, we analyzed the expression of *sunA*-GFP or *sunI*-GFP promoter fusions in several mutant backgrounds. This allowed us to delineate the different factors regulating *sunA*. Here we show that while multiple regulators influence the levels of *sunA* expression, the heterogeneity of the *sunA* promoter (P*sunA*) activity is controlled by only three of them, namely Spo0A, AbrB and Rok. Conversely, through analysis of the *sunI*-GFP promoter fusion we found that the immunity gene *sunI* is homogeneously expressed in all cells of the investigated population, which will protect them from the toxic effects of sublancin.

## Materials and Methods

### Strains, plasmids and primers

The bacterial strains and plasmids used in this study are listed in Table 1. Primers used for creating *sunI*, *rok* or *abh* mutations are listed in Table 2. *Escherichia coli* strains were grown in Lysogeny Broth (LB) at 37 °C with vigorous shaking or on LB agar plates. For standard laboratory practices *B*. *subtilis* was grown in either LB broth at 37 °C with vigorous shaking or on LB agar plates. *B*. *subtilis* was transformed using a standard transformation procedure with plasmid or chromosomal DNA (isolated as described by Bron & Venema, 1972)^31^ using Paris Medium consisting of 10.7 mg/ml K_2_HPO_4_, 6 mg/ml KH_2_PO_4_, 1 mg/ml trisodium citrate, 0.02 mg/ml MgSO4, 1% glucose, 0.1% casamino acids (Difco), 20 μg/ml L-tryptophan, 2.2 μg/ml ferric ammonium citrate and 20 mM potassium glutamate^19^. Growth media were supplemented with antibiotics where appropriate; ampicillin (Ap) 100 μg/ml, chloramphenicol (Cm) 5 μg/ml, erythromycin (Em) 1 μg/ml, kanamycin (Km) 20 μg/ml, phleomycin (Phleo) 4 μg/ml, spectinomycin (Sp) 100 μg/ml. 0.5 mM IPTG was added to induce expression of the *sad67* allele. Deletion mutants created in this study were constructed as described by Tanaka *et al*., and introduced into the genome of strain 168^32^. Mutations were confirmed by PCR and functional screens for competence and enzyme secretion. Promoter-GFP fusions used in this study are single copy chromosomal insertions. Integration of the promoter-GFP fusions into the genome was achieved via single crossover recombination using the pBaSysBioII plasmid^33^. This plasmid cannot replicate in *B*. *subtilis*, ensuring the presence of only a single copy of the promoter-GFP fusion in the chromosome, which precludes unwanted gene dosage effects. The P*sunI*-GFP promoter fusion was constructed as described by Botella *et al*.^33^ using the P*sunI* primers presented in Table 2.

**Table 1:** *B*. *subtilis* strains and plasmids.

Strains/genotype	Relevant properties	Reference
168 *trpC2*	parental strain used in this study; produces sublancin 168	Kunst *et al*.^44^
ΔSPβ	SPβ prophage deletion mutant; sublancin sensitive	Dorenbos *et al*.^7^
P*sunA*-GFP	Carries a *sunA* promoter GFP fusion created with pBaSysBioII; Sp^r^	Piersma *et al*.^36^
P*sunI*-GFP	Carries a *sunI* promoter GFP fusion created with pBaSysBioII; Sp^r^	This work
*sad67*	IPTG-inducible Spo0A-P production; Cm^r^	Ireton *et al*.^37^
*sad67* P*sunA*-GFP	IPTG-inducible Spo0A-P production; Cm^r^, Sp^r^	This work
*sad67* P*sunI*-GFP	IPTG-inducible Spo0A-P production, Cm^r^, Sp^r^	This work
Δ*spo0A*	Km^r^	Boonstra *et al*.^45^
Δ*spo0A* P*sunA*-GFP	Km^r^, Sp^r^	This work
Δ*clpX*	Cm^r^	Wiegert and Schumann 2001^46^
Δ*clpX* P*sunA*-GFP	Cm^r^, Sp^r^	This work
Δ*abbA*	Km^r^	Blencke *et al*.^47^
Δ*abbA* P*sunA*-GFP	Km^r^, Sp^r^	This work
Δ*yjbH*	BFA2867, Em^r^	Kobayashi *et al*.^48^
Δ*yjbH* P*sunA*-GFP	BFA2867, Em^r^, Sp^r^	This work
Δ*sigX*	HB10103, Km^r^	Luo & Helmann, 2009^21^
Δ*sigX* P*sunA*-GFP	HB10103, Km^r^, Sp^r^	This work
Δ*sigM*	HB10016,	Luo & Helmann, 2009^21^
Δ*sigM* P*sunA*-GFP	HB10016, Tc^r^, Sp^r^	This work
Δ*sigXM*	Km^r^, Tc^r^	This work
Δ*ccpA*	GP302, Em^r^	Ludwig *et al*.^49^
Δ*ccpA* P*sunA*-GFP	GP302, Em^r^, Sp^r^	This work
Δ*spx*	TR2, Cm^r^	Rochat *et al*.^27^
Δ*spx* P*sunA*-GFP	TR2, Cm^r^, Sp^r^	This work
Δ*abrB*	TMB082, Tc^r^	Jordan *et al*.^50^
Δ*abrB* P*sunA*-GFP	TMB082, Sp^r^	This work
Δ*abrB* P*sunI*-GFP	TMB082, Sp^r^	This work
Δ*abh*	Phleo^r^	This work
Δ*abh* P*sunA*-GFP	Phleo^r^, Sp^r^	This work
Δ*rok*	Phleo^r^	This work
Δ*rok* P*sunA*-GFP	Phleo^r^, Sp^r^	This work
Δ*rok* Δ*abrB* P*sunA*-GFP	Phleo^r^, Tc^r^, Sp^r^	This work
Δ*rok sad67* P*sunA*-GFP	Phleo^r^, Cm^r^, Sp^r^	This work
Δ*rok* P*sunI*-GFP	Phleo^r^, Sp^r^	This work
Δ*comA*	Cm^r^	Guillen *et al*.^51^
Δ*comA* P*sunA*-GFP	Cm^r^, Sp^r^	This work
Δ*yvrGH*	Km^r^	Serizawa *et al*.^25^
Δ*yvrGH* P*sunA*-GFP	Km^r^, Sp^r^	This work
**Plasmid name**	**Relevant properties**	**Reference**
BaSysBioII	LIC cloning vector for creating GFP promoter fusions.	Botella *et al*.^33^
P*sunA*-GFP	*sunA* promoter region cloned into BaSysBio II	Piersma *et al*.^36^
P*sunI*-GFP	*sunI* promoter region cloned into BaSysBio II	This work

**Table 2:** Primers used in this study to create *sunI*, *rok*, or *abh* mutations.

PsunI-GFP Fwd	CCGCGGGCTTTCCCAGCcgtacacataataaagttgg
PsunI-GFP Rev	GTTCCTCCTTCCCACCgtttttatataattttaccatg
Rok P1	Gttttgaaatggaagcagtc
Rok P2	CGACCTGCAGGCATGCAAGCTtcctcaatgtaccccctatc
Rok P3	CGAGCTCGAATTCACTGGCCGTCGatataaagaaaaactgcttgg
Rok P4	cttctcagaaagctgatcgt
Abh1	GAAGCAAGAAATTTGCCGCGT
Abh 2	CGACCTGCAGGCATGCAAGCTAACATTTAAAGGAAGAAGGGTTTTT
Abh 3	CGAGCTCGAATTCACTGGCCGTCGaaagaaacatttaaaggaagaagggtttttAATTATGCTAAAAAAGGCGGAGT
Abh 4	CTCAAACAAATGGGAAGTCC

The possible loss of the P*sunA*-GFP fusion from *B*. *subtilis* 168 upon overnight culturing in the absence of selective antibiotic pressure was tested by plating 100 µl aliquots of 1000x and 10000x dilutions of the culture on LB-agar without antibiotics. Upon overnight incubation of the plates, the colonies were imaged for GFP fluorescence on an Amersham Typhoon imager, and the fluorescence intensities of all colonies on a plate were quantified using ImageJ.

### Sublancin susceptibility

Sublancin susceptibility assays were carried out as previously described with minor modifications^7,34^. A 1:100 dilution of an overnight culture of *B*. *subtilis* 168∆SPβ was plated either on regular (full-strength) LB agar medium or, for comparison with microscopy-derived results, on four-fold diluted LB medium containing 1% NaCl and 1.5% agarose (quarter-strength LB medium) in a Petri dish. This created a lawn of sublancin-susceptible cells onto which 1 µl of an overnight culture of a sublancin-producing strain was spotted. After overnight incubation at 37 °C, the Petri dish was photographed and halo- and colony diameters in the images were quantified using ImageJ. Since sublancin is distributed over a surface, the diameters of colony and halo were used to calculate the halo surface area. Halo surface sizes of mutant producer strains were normalized against that of the wild-type strain to enable comparisons to promoter activity assays.

### Microtiter plate experiments

Strains containing the P*sunA*-GFP fusion were grown overnight in LB. The next day the strains were diluted 1:200 in the same medium and grown for 2.5 hours.

$${\rm{d}}={({{\rm{OD}}}_{{\rm{977}}}-{{\rm{OD}}}_{{\rm{900}}})}_{{\rm{sample}}}/{{(\mathrm{OD}}_{{\rm{977}}}-{{\rm{OD}}}_{{\rm{900}}})}_{{\rm{reference}}}\times \,{\rm{1}}\,{\rm{cm}}{\rm{.}}$$

OD_600_ values of each sample were divided by the path length.

To compare data from microscopy experiments with data from microtiter plate experiments, the LB was diluted to quarter strength and the NaCl corrected to the standard 1%.

### Time-lapse microscopy

Agarose slides were prepared as described by Botella *et al*.^33^, using quarter-strength LB with 1.5% agarose (Merck). To prepare bacteria for time-lapse microscopy, strains were grown overnight in LB medium containing the appropriate antibiotics. After a 1:200 dilution in quarter-strength LB medium, the strains were grown for 2.5 h. Next, the strains were spotted onto a 1 to 1.5 mm wide strip of agarose on the prepared slide. A time-lapse movie of the growing bacteria was recorded using a Leica DM 5500 B microscope. Phase contrast and fluorescence images were recorded every 5 to 7 minutes, depending on the number of samples. Fluorescence pictures were recorded using a Leica EL6000 lamp with an L5 filter cube. Both the lamp intensity and the attenuator inside the microscope were set to 10% to minimize phototoxicity.

### Image analysis

Data was extracted from recorded images using FIJI, an ImageJ-based software package^35^ (http://pacific.mpi-cbg.de/wiki/index.php/Fiji), which is freely available. Cellular fluorescence and GFP expression heterogeneity were measured with the TLM-Quant pipeline as described previously^36^. This method consists of two types of visualizations of the data: 1. Expression heterogeneity expressed as standard deviation in cellular fluorescence, to enable easy comparison of different samples. 2. A 3D histogram to determine if bistability exists within the samples that are found to display expression heterogeneity. All experiments were carried out in duplicate. Briefly, the following properties were extracted from the images that were obtained in the microscopy analysis. Cell outlines were derived from phase contrast images and used to distinguish cells in the fluorescent channel. From individual cells, fluorescence values were obtained, which were then corrected for background fluorescence. All cells combined were considered a population, from which a mean value and a standard deviation (SD) were calculated. The SD was used as a measure for heterogeneity, and was corrected for the SD of fluorescence caused by external factors. An inducible P*spac*-GFP fusion strain was used as a control for homogeneous promoter activity that allowed a correction of the SD in fluorescence. To this end, the strain was induced with four different concentrations of isopropyl β-D-1-thiogalactopyranoside (IPTG) and the obtained SD values of all data points were combined in one figure, where the average fluorescence was plotted on the X-axis and the SD on the Y-axis. A trend-line was drawn through these points, showing that a linear correlation exists between fluorescence intensity and inherent heterogeneity. The equation of this line was subtracted from the calculated heterogeneity values, to correct for heterogeneity that is inherent to the microscopy setup and the detected GFP transcription. After correction, the coefficient of variation was calculated as the ratio between the SD in fluorescence intensity and the mean fluorescence. In this assay, a high heterogeneity value is an indicator that can reflect two different situations, namely: i. a higher SD in cellular fluorescence, which originates from a higher number of fluorescent cells, or ii, an increased intensity of existing fluorescent cells.

## Results and Discussion

### Expression of *sunA* and production of active sublancin on LB are determined by the transcriptional regulators Abh, AbrB, Rok, Spo0A, σ^M^ and σ^X^

Active sublancin was previously shown to be produced at the end of the exponential growth phase and throughout stationary phase^34^. To calibrate our experimental set-up, we analyzed the promoter activity of *sunA* using a GFP promoter fusion and time-lapse microscopy with cells growing on LB agar. Notably, LB was selected for these analyses, because little if any active sublancin is produced when cells are grown on minimal media within the 17 h time frame of our experiments. This is in line with previous findings of van der Donk *et al*.^8^, who reported production of active sublancin on M9 minimal medium starting 48 h after inoculation and reaching an optimum at 60–72 hours. Thus, the onset in the secretion of active sublancin on minimal medium is relatively late by *Bacillus* standards, and cannot be captured in our time-lapse microscopy setup.

**Figure 2:**
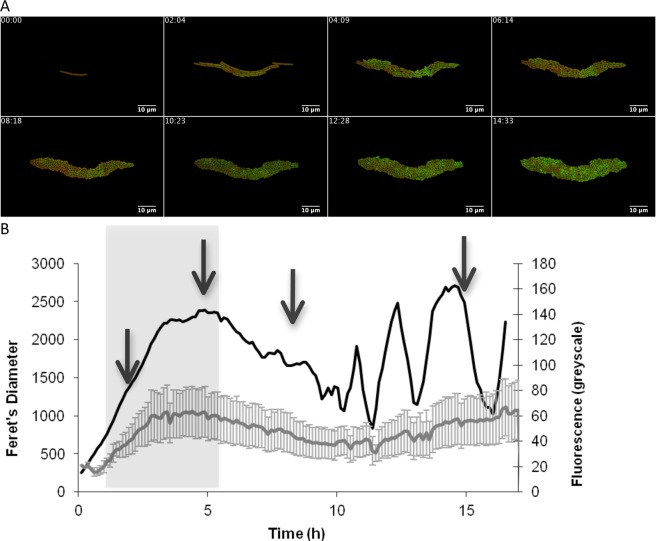
Expression of *sunA* as determined by time-lapse fluorescence microscopy. (**A**) Montage of time-lapse fluorescence microscopy images of *B*. *subtilis* 168 containing a *PsunA* promoter*-*GFP fusion and grown on LB. The time points at which images were captured are shown. (**B**) Growth curve and fluorescence of *B*. *subtilis* 168 *PsunA-*GFP as derived from the complete analysis for which selected images are shown in panel A. The cumulative feret’s diameter represented by the black line was used as a measure for growth (black line). The mean fluorescence at each time point is represented by a grey line and the error bars represent the level of fluorescence heterogeneity. The arrows indicate sample points for quantification of the fluorescence heterogeneity as shown in Fig. 3. Fluorescence recordings in the grey zone were used to determine the maximum promoter activity as shown in Fig. 3.

When the cells were grown on LB, a low level of GFP expression was observed during the exponential phase of growth before the promoter became highly active during stationary phase (Fig. 2). As shown by the size of the error bars in the recorded fluorescence in Fig. 2B, there was a substantial heterogeneity in GFP fluorescence. This expression behavior is comparable to a previous expression analysis of the *sunA* promoter^30^ and we therefore concluded that our set-up was suitable for a dissection of factors involved in the heterogeneous expression of *sunA*. Of note, we verified that the observed heterogeneity in GFP fluorescence was not due to loss of the integrated pBaSysBioII plasmid used to generate the *sunA*-GFP fusion by plating bacteria cultured overnight in the absence of selective antibiotic pressure and quantifying the fluorescence intensities of the resulting colonies. The fluorescence intensities in the histogram in Supplementary Fig. S1 show that all plated cells were fluorescent and, thus, had retained the integrated plasmid.

**Figure 3:**
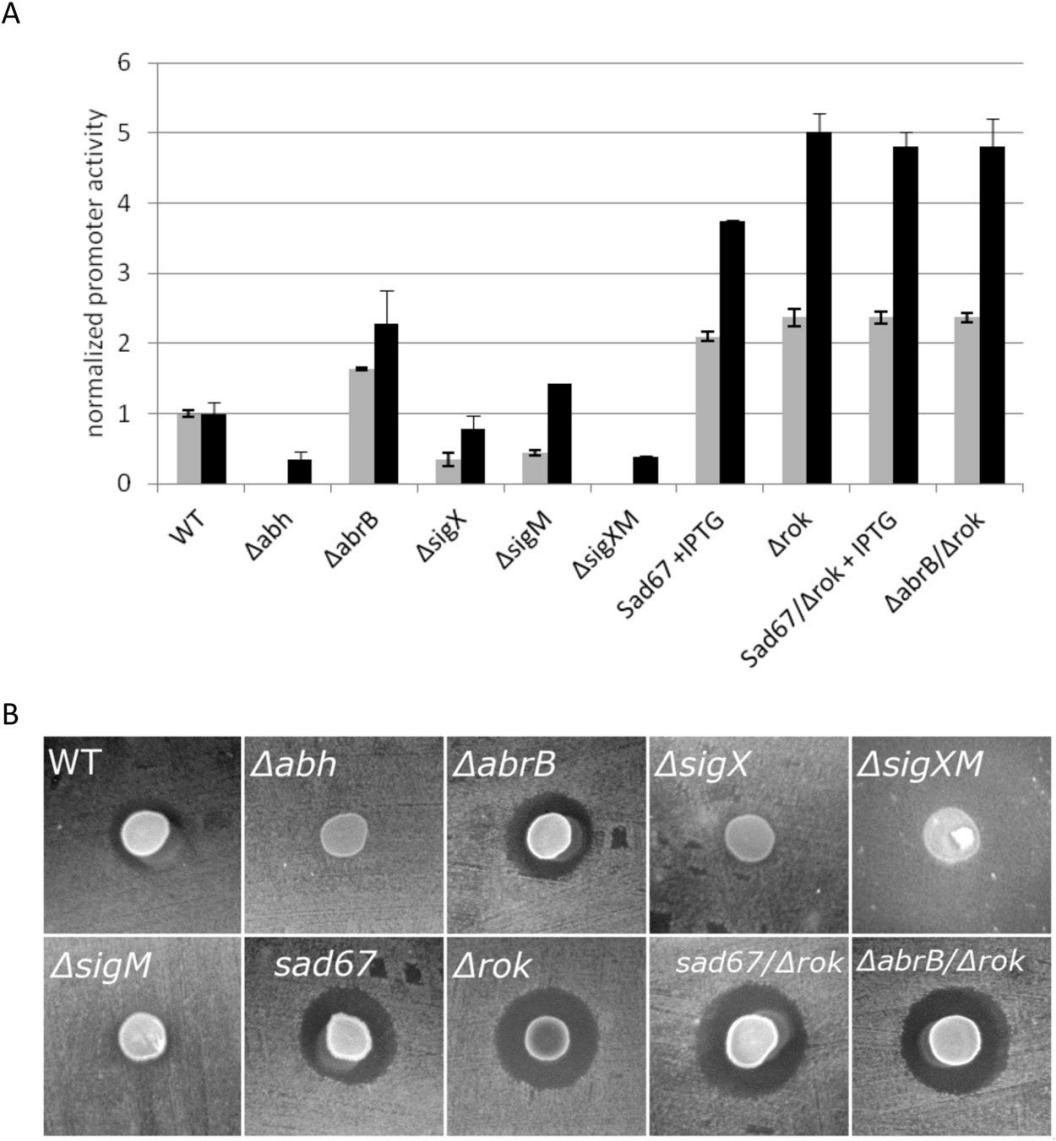
Correlation of *sunA* promoter activity with the production of active sublancin. (**A**) Comparison of the maximum *sunA* promoter activity (black bars) to the production of active sublancin (grey bars). The maximum *sunA* promoter activity values for each investigated mutant strain were determined by time-lapse fluorescence microscopy as in Fig. 2, and normalized against the maximum promoter activity determined for the parental strain 168 (WT). The production of active sublancin by each investigated mutant strain was determined by measuring the surface of sublancin-induced growth inhibition zones on a lawn of sublancin-susceptible cells of the *B*. *subtilis* ΔSPβ strain as shown in panel B. The sublancin production by each strain was then normalized against that of the parental strain 168. (**B**) Representative images for growth inhibition as caused by sublancin-producing cells that were spotted on a lawn of *B*. *subtilis* ΔSPβ. Mutations in the genome of sublancin-producing cells are marked in each image. WT, *B*. *subtilis* 168.

Several transcriptional regulators have been shown to influence the expression of *sunA*. From a literature-based search, we deduced that the regulators AbbA, Abh, AbrB, CcpA, ClpX, ComA, Rok, SigM, SigX, Spo0A, Spx, YjbH, YvrGH have been associated with changes in the expression level of *sunA*. Accordingly, we deleted the respective genes from the genome of the strain expressing the *sunA*-GFP promoter fusion (Table 1). Next, we carried out expression analyses by monitoring the levels of GFP produced in these strains during growth on LB. Under the tested conditions, no significant effects on the activity of P*sunA*-GFP were observed when the *ccpA*, *comA*, *spx*, or *yvrGHb* genes were deleted (data not shown). This relates, most likely, to differences in the growth conditions applied here and in previous studies that had implicated the respective regulators in *sunA* expression. On the other hand, clear effects on the activity of P*sunA*-GFP and the production of active sublancin were observed in strains with mutations in the *abh*, *abrB*, *sigM*, *sigX*, *spo0A* or *rok* genes (Fig. 3).

Previous investigation of the regulation of *sunA* expression showed that regulators of the entry into stationary phase play a role in the production of sublancin^21^. Indeed, deletion of *spo0A* resulted in a complete elimination of *sunA* transcription and sublancin production (Supplementary Fig. S2). Conversely, induction of the *sad67* allele of *spo0A*, which leads to the Spo0A pathway being permanently activated^37^, resulted in very high GFP expression from the P*sunA*-GFP promoter fusion and strongly enhanced production of active sublancin (Fig. 3). One of the main transcriptional rearrangements caused by activation of the Spo0A pathway is the blocking of *abrB* transcription. Deletion of *abrB* from the *B*. *subtilis* genome resulted in enhanced levels of transcription from the *sunA* promoter and, accordingly, enhanced production of active sublancin (Fig. 3). Abh is a paralogue of AbrB that functions by binding to many of the same transcription factor binding sites as AbrB. Notably, Abh was previously shown to activate transcription of *sunA*^38^ and, indeed, the deletion of *abh* caused a severe down-regulation of the transcription from the *sunA* promoter as well as sublancin production (Fig. 3). Expression of *abh* is regulated by two ECF sigma factors, σ^M^ and σ^X^. While individual deletions of the *sigM* or *sigX* genes had less dramatic effects on P*sunA* activity and sublancin production than the *abh* deletion, combining the *sigM* and *sigX* deletions in one strain resulted in a severe reduction in the activity of P*sunA* and the production of sublancin to similar levels as observed for the *abh* mutant strain (Fig. 3). Intriguingly, significant *sunA* expression was observed in the *sigM* mutant strain, whereas only marginal production of active sublancin was detectable. This would suggest that SigM impacts on expression of a gene needed for the production of active sublancin. Altogether, our present observations were in good agreement with the previous findings of Luo & Helmann^21^ as represented in Fig. 1. Notably, AbrB is also post-translationally controlled by AbbA^39^ and, accordingly, deletion of the *abbA* gene resulted in a reduction in transcription from the *sunA* promoter (Supplementary Fig. S3). Lastly, during exponential growth the transcriptional repressor Rok is under the control of AbrB^40^. Rok has been shown to bind to the promoter region of *sunA*^23,24^ and we therefore also assessed the influence of a *rok* deletion on P*sunA*-GFP activity and sublancin expression. Indeed, the *rok* deletion did increase the *sunA* promoter activity and sublancin expression. In fact, this deletion resulted in the highest levels of P*sunA*-GFP and sublancin activity observed in the present studies (Fig. 3). Furthermore, the effect of the *rok* deletion was dominant over the effects of induction of the *sad67* allele of *spo0A* or the deletion of *abrB* (Fig. 3). Together, these findings show that Abh, AbrB, Rok, Spo0A, σ^M^ and σ^X^ are the key determinants for *sunA* expression and production of active sublancin in *B*. *subtilis* cells growing on LB.

### AbrB, Rok and Spo0A determine *sunA* expression heterogeneity

**Figure 4:**
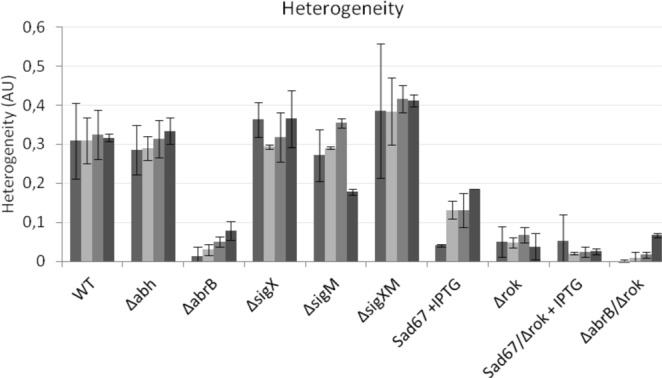
Quantification of *sunA* expression heterogeneity. Heterogeneity in the expression of the *sunA* promoter-GFP fusion in growing cells of various mutant strains and the parental strain 168 (WT) was assessed by time-lapse fluorescence microscopy. GFP expression heterogeneity was assessed at different time points along the growth curve as marked by arrows in Fig. 2. Correspondingly, the differently grey-shaded bars indicated for each strain represent, from left to right, *sunA* expression heterogeneity in the exponential growth phase, the transition phase, the early stationary phase and the late stationary phase. Heterogeneity values were calculated as the mean variance in cellular GFP fluorescence (indicated in arbitrary units, AU).

While *sunA* has previously been shown to be expressed heterogeneously, the regulatory basis for this heterogeneous expression has not yet been established. To identify the origin of the observed *sunA* expression heterogeneity, we quantified this heterogeneity in *abh*, *abrB*, *rok*, *spo0A-sad67*, *sigM* or *sigX* mutant strains with the P*sunA*-GFP fusion using the previously developed TLM-Quant pipeline^36^. Specifically, the heterogeneity in *sunA* expression was measured at four different time points across the growth curve, namely at mid-exponential growth, transition stage, and two late stages when large microcolonies had been established (Figs 2 and 4). As shown in Fig. 4, the *sunA* expression heterogeneity was neither influenced by individual deletion of the *abh*, *sigX* or *sigM* genes, nor the deletion of both *sigX* and *sigM*. On the other hand, the *sunA* expression heterogeneity was strongly reduced by deletion of *abrB* or *rok* and the induced expression of the *sad67* allele of *spo0A*. In the first place, these findings show that AbrB, Rok and Spo0A are the key determinants for *sunA* expression heterogeneity. A second important conclusion is that *sunA* expression heterogeneity is not strictly related to the level of P*sunA* promoter activity. In particular, while P*sunA* promoter activity in the *abh* or *sigX* and *sigM* mutants was very low, the *sunA* expression heterogeneity in these mutant strains was very similar to that observed in the parental strain 168. Conversely, while the P*sunA* promoter activity was at the highest level in strains lacking *abrB* and/or *rok*, these strains showed the lowest levels of *sunA* expression heterogeneity. Overall the results show that high-level expression of *sunA* is accompanied by relatively low expression heterogeneity, whereas lower-level *sunA* expression is accompanied by relatively high expression heterogeneity (Fig. 4).

The induced expression of the *sad67* allele of Spo0A resulted in a ~3.5-fold increase in the *sunA* promoter activity and a ~2-fold increase in production of active sublancin while, at the same time it caused a ~2- to 6-fold reduction in *sunA* expression heterogeneity depending on the growth stage. Spo0A-Sad67 is a constitutively active mutant form of Spo0A that mimics the behavior of Spo0A-P^37^. Spo0A is well studied for its role in the bistable process of sporulation. In this complex regulatory cascade, the sporulation phosphorelay switching mechanism transfers a phosphate group to Spo0A to form the regulatory active Spo0A-P^41,42^. Expression of Spo0A is under the influence of several positive feedback loops and these auto-inducing loops cause the bistable phosphorylation of Spo0A. This mechanism ensures on-off switching of the sporulation phenomenon. Unfortunately, our experimental setup does not allow conclusions on a possible bistable expression of the *sunA*-GFP fusion by distinct sub-populations of the cells in the presence or absence of Spo0A-Sad67 induction. Nevertheless, Spo0A remains required for *sunA* expression under these conditions as the deletion of *spo0A* completely blocked P*sunA* promoter activity (Supplementary Fig. S2).

AbrB and Rok are under the direct influence of Spo0A, and our experiments show that they are key factors not only controlling the *sunA* expression level and consequently production of active sublancin, but also the heterogeneity of expression of P*sunA-*GFP. The heterogeneity in the *abrB* and *rok* mutants was reduced ~6-fold compared to the wild-type strain and combining these two mutations reduced this even further to barely detectable levels for most growth stages (Fig. 4). Interestingly, although deletion of *rok* or *abrB* resulted in similar levels of heterogeneity, the *rok* deletion caused a rise in P*sunA* promoter activity that was 2-fold higher than in the *abrB* mutant. The production of active sublancin in the *rok* deletion mutant was ~1.6 times higher than in the *abrB* deletion strain (Figs 3 and 4). The Abh protein is known to compete with AbrB for binding to the promoter region of *sunA* to induce its expression^22^. Consistent with this AbrB-antagonizing effect of Abh, the deletion of *abh* from the genome resulted in a ~3-fold reduction in *sunA* expression and a failure to produce active sublancin, whereas the level of heterogeneity in *sunA* expression was not altered. Similarly, the levels of promoter activity and sublancin production were reduced in the sigma factor mutants but, in this case, the *sunA* expression heterogeneity was maintained. A *sigX sigM* double mutant behaved very similarly to an *abh* mutant in all respects, which is consistent with the requirement of σ^X^ and σ^M^ for *abh* expression (Figs 1,3 and 4).

Taken together, the present results suggest that the interplay between Spo0A-P, AbrB and Rok is crucial for generating heterogeneity in *sunA* expression when cells are grown on LB. We note that the inhibitory effects of the *sunA* repressors AbrB and Rok are not equal in all cells and that the majority of *sunA* expression heterogeneity is removed upon deletion of their genes. The deletion of *rok* had the strongest effect on the *sunA* promoter, but since AbrB is also known to repress the expression of *rok*, increased levels of Rok are probably present in the *abrB* mutant cells, which is likely to account for the differences in the levels of active sublancin produced by the *abrB* and *rok* mutant strains. It seems therefore that the generation of *sunA* expression heterogeneity is due to a balance in the level of phosphorylation of Spo0A, which upon phosphorylation acts to repress the expression of *abrB* and *rok*. This potential balance between the regulation of the transcription factors generating *sunA* expression heterogeneity is highlighted by the dramatically reduced heterogeneity in *sunA* expression in the *abrB rok* double mutant. Lastly, we should point out that, by creating the *sunA* promoter GFP fusion through single cross-over integration of the pBaSysBioII plasmid into the *sunA* locus, the *sunA* regulatory sequences were duplicated. This could potentially influence the effective level of *sunA* regulators due to a dilution effect. On the other hand, the presently followed approach precludes potentially deleterious polar effects on the expression of downstream genes in the *sunA* operon (i.e. *sunT*, *bdbA*, *sunS* and *bdbB*), which would have prevented the combined analysis of *sunA* expression at the single-cell level and assessment of the overall levels of sublancin production in one and the same system. Further, in the context of our single cell GFP expression analyses, it is important to bear in mind that upon *sunA* repression, there may be the same level of heterogeneity in the bacterial population, but a lower mean GFP level. This relates to the possibility that, if fewer cells at any given moment have the *sunA* promoter switched on, these cells still need to dilute the already synthesized GFP molecules, which could lead to heterogeneity in the population’s GFP levels. Instead, when *sunA* repression is relieved, most cells will turn the *sunA* promoter on and, accordingly, the mean fluorescence will go up while heterogeneity in the GFP level may decrease.

### *sunI* expression is homogenous throughout the cell population

**Figure 5:**
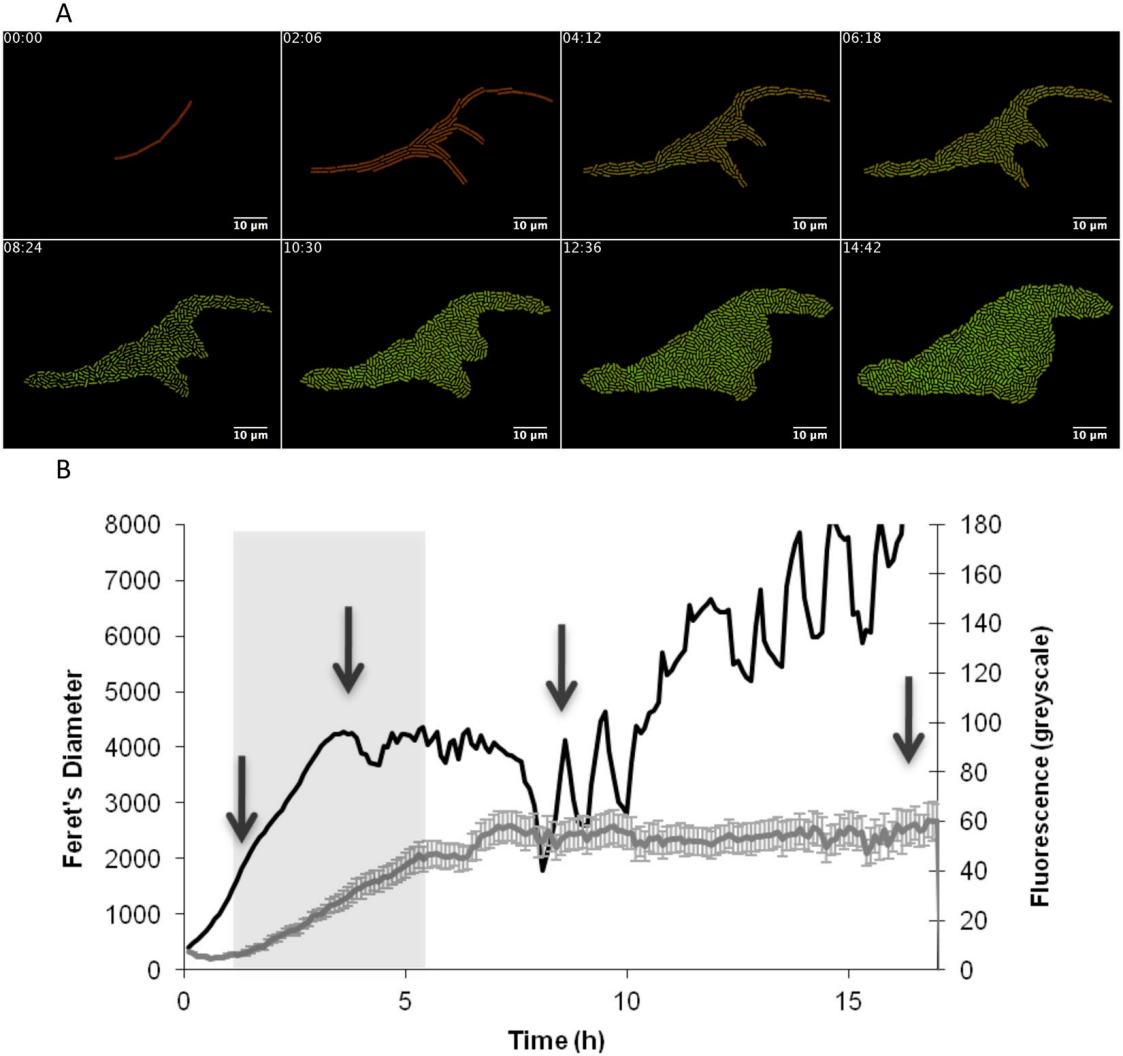
Expression of *sunI* in growing cells as determined by time-lapse fluorescence microscopy. (**A**) Montage of time-lapse fluorescence microscopy images of *B*. *subtilis* 168 containing a *PsunI* promoter-GFP fusion and grown on LB. The time points at which images were captured are shown. (**B**) Growth curve and fluorescence of *B*. *subtilis* 168 P*sunI*-GFP as derived from the complete analysis for which selected images are shown in panel A. The cumulative feret’s diameter represented by the black line was used as a measure for growth (black line). The mean fluorescence at each time point is represented by a grey line and the error bars represent the level of fluorescence heterogeneity. The arrows indicate sample points for quantification of the fluorescence heterogeneity as shown in Fig. 6. Fluorescence recordings in the grey zone were used to determine the maximum promoter activity as shown in Fig. 6.

**Figure 6:**
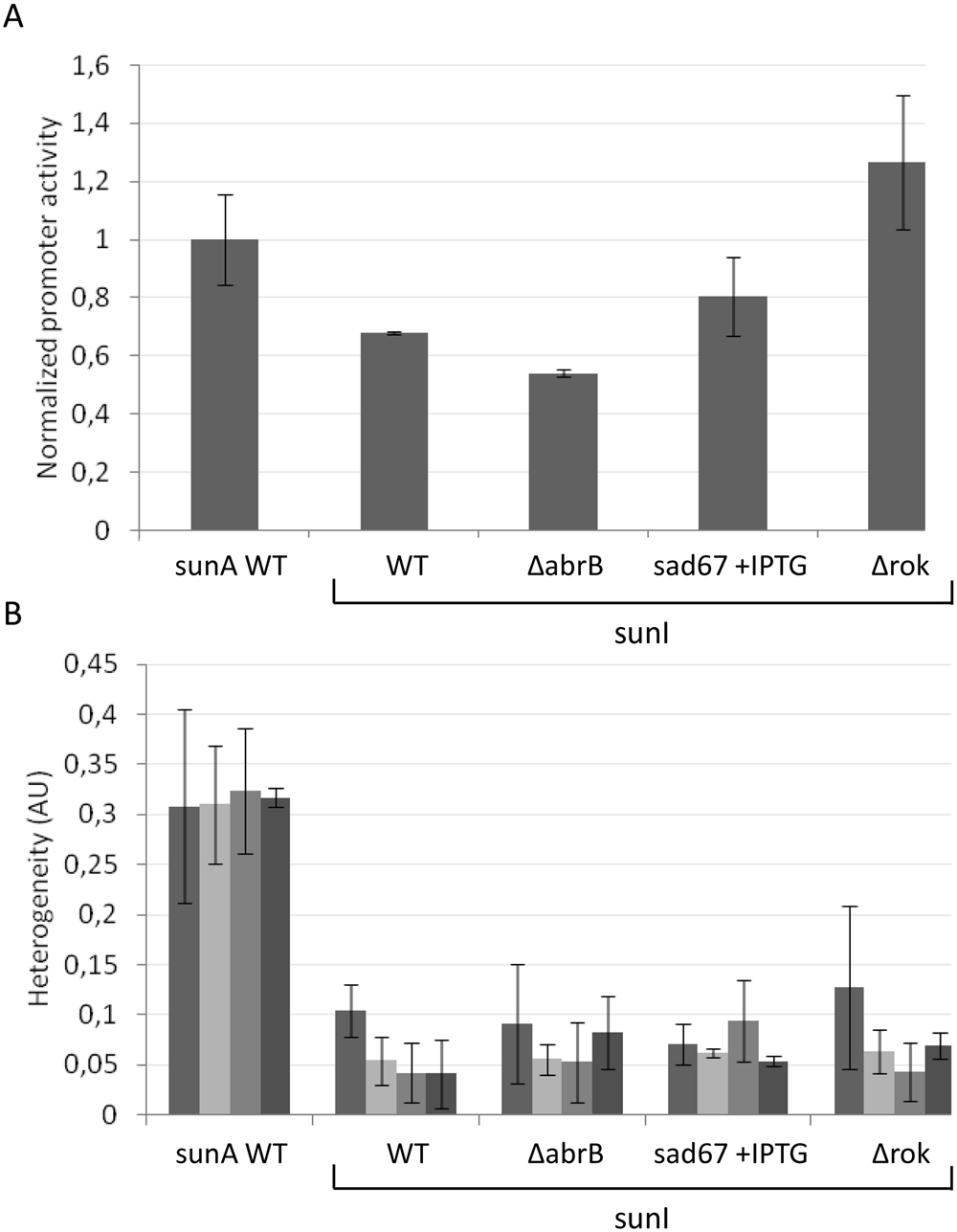
Comparison of the promoter activity and expression heterogeneity of *sunA* and *sunI* in growing cells. (**A**) Comparison of the maximum *sunI* promoter activity in various mutant strains to the maximum promoter activity of *sunA* in cells of the parental strain *B*. *subtilis* 168 (WT). The maximum *sunI* promoter activity values for each investigated mutant strain were determined by time-lapse fluorescence microscopy as in Fig. 5, and normalized against the maximum promoter activity determined for the parental strain 168 (WT). (**B**) Comparison of the expression heterogeneity of various strains expressing a *sunI* promoter-GFP fusion to the expression heterogeneity determined for the *sunA* promoter-GFP fusion in the parental strain 168 (WT). GFP expression heterogeneity was assessed at different time points along the growth curve as marked by arrows in Fig. 5. Correspondingly, the differently grey-shaded bars indicated for each strain represent, from left to right, *sunA* or *sunI* expression heterogeneity in the exponential growth phase, the transition phase, the early stationary phase and the late stationary phase. Heterogeneity values were calculated as the mean variance in cellular GFP fluorescence (indicated in arbitrary units, AU).

The *sunI* gene encodes the immunity protein for sublancin, and all cells producing active sublancin must express this gene to be immune to the effects of this bacteriocin. However, it was not known to date whether *sunI* expression would be heterogeneous, following the heterogeneous expression of *sunA*, or whether *sunI* expression would be homogeneous throughout the population. We therefore assessed the expression of *sunI* in cells growing on LB and expressing a P*sunI* promoter GFP fusion. As evidenced by time-lapse fluorescence microscopy (Fig. 5), *sunI* expression remained highly homogeneous over all stages of the growth curve. Notably, the activity of the *sunI* promoter was only slightly lower than that of the *sunA* promoter and it was only mildly influenced by deletions of *abrB* or *rok* (Fig. 6A). Induced expression of the *sad67* allele of *spo0A* had no significant effect on *sunI* promoter activity. Importantly, none of the mutations that had major effects on the heterogeneous expression of *sunA* had a significant effect on the very low level of heterogeneity in the expression of *sunI*. In fact the expression heterogeneity of *sunI* in the parental strain 168 was within the same range as that determined for *sunA* in mutants lacking the *abrB* or *rok* genes. It can thus be concluded that *sunI* is very homogeneously expressed in cells growing on LB.

Altogether, the present findings show that the expression of *sunA* and *sunI* is very differently regulated. This is consistent with the studies by Nicolas *et al*.^2^, where the overall expression level of *sunA* was shown to be highly variable depending on the growth condition studied. In contrast, the variability in expression level of *sunI* was relatively small across the 104 conditions tested, which seems to suggest that *sunI* is less susceptible to transcriptional regulation than *sunA*. The strong involvement of Rok in *sunA* regulation is noteworthy in this context, since Rok is a negative regulator of genes involved in horizontal gene transfer. Rok inhibits uptake of foreign DNA by inhibiting the main transcription factor of competence, ComK^40^, and it also represses the transcription of genes in A + T rich regions of the *B*. *subtilis* chromosome, which are the result of horizontal gene transfer^24^. The SPβ prophage, and therefore *sunA* and *sunI*, are A + T rich and have been acquired by *B*. *subtilis* through horizontal gene transfer. Thus, one would expect not only *sunA*, but also *sunI* to be a target for Rok regulation. However, the marginal influence of Rok on *sunI* expression as observed in the present study suggests that the regulation of this gene by Rok was minimized to ensure optimal sublancin immunity of the cells that contain the SPβ prophage.

## Conclusions

Here we describe the differential regulation of genes encoding for the bacteriocin sublancin and its cognate immunity protein. Only part of the *B*. *subtilis* population expresses *sunA* at maximum level. However, the whole population can benefit from this high-level expression by creating an environment in which *B*. *subtilis* is able to kill competitors, leaving more nutrients for itself. On the other hand, for the whole isogenic population to survive, the immunity protein must be expressed continuously by all cells. This apparently placed a strong selective pressure on the promoter of *sunI* to remain consistently stable within the population, but it may have allowed the *sunA* promoter to evolve to become growth phase-dependently and heterogeneously expressed. Heterogeneous production of sublancin could be beneficial for the population since only a small number of producers are required to provide bactericidal activity. Other members of the bacterial colony would then have more resources available for other processes. Here it is noteworthy that the *sunA* gene is amongst the most highly expressed genes of *B*. *subtilis*. Thus, producing sublancin is likely to be ‘expensive’ to the cell, especially since at least four additional proteins (*i*.*e*. SunT, SunS, BdbB and SunI) are needed to secrete active sublancin and since protein synthesis is a resource-costly process^43^. In this context, the timing of *sunA* gene expression seems optimal as the production of sublancin is likely most beneficial during late exponential phase and stationary phase when nutrients become limited. Its production would not only give the producing cells a competitive advantage over other species in the vicinity, but also release additional nutrients due to the death of such competitors. Lastly, constitutive *sunI* expression will not only provide a competitive advantage to cells in the sublancin-producing population, but also to the SPβ prophage, which ensures in this way that it is stably maintained in the *B*. *subtilis* genome. Clearly, cells that would lose the SPβ prophage would become susceptible to sublancin and therefore die. Altogether, this suggests a mutualistic evolutionary strategy entertained by the SPβ prophage and its *Bacillus* host, ensuring both stable prophage maintenance and a maximal competitive advantage for the host at minimal costs.

## Supplementary information

Supplementary Figures S1, S2 and S3

## Data Availability

All data related to this manuscript are available.

## References

[1] Buescher JM (2012). Global network reorganization during dynamic adaptations of *Bacillus subtilis* metabolism. Science.

[2] Nicolas P (2012). Condition-dependent transcriptome reveals high-level regulatory architecture in *Bacillus subtilis*. Science.

[3] Radeck J, Fritz G, Mascher T (2017). The cell envelope stress response of *Bacillus subtilis*: from static signaling devices to dynamic regulatory network. Current Genetics.

[4] Völker U, Hecker M (2005). From genomics via proteomics to cellular physiology of the Gram-positive model organism *Bacillus subtilis*. Cellular Microbiology.

[5] Abriouel H, Franz CMAP, Omar NBen, Galvez A (2011). Diversity and applications of *Bacillus* bacteriocins. FEMS Microbiol. Rev..

[6] Stein T (2005). *Bacillus subtilis* antibiotics: Structures, syntheses and specific functions. Molecular Microbiology.

[7] Dorenbos R (2002). Thiol-disulfide oxidoreductases are essential for the production of the lantibiotic sublancin 168. J. Biol. Chem..

[8] Oman TJ, Boettcher JM, Wang H, Okalibe XN, Van Der Donk WA (2011). Sublancin is not a lantibiotic but an S-linked glycopeptide. Nat. Chem. Biol..

[9] Stepper J (2011). Cysteine S-glycosylation, a new post-translational modification found in glycopeptide bacteriocins. FEBS Lett..

[10] Biswas SGD, Gonzalo CV, Repka LM, Van Der Donk WA (2017). Structure-Activity Relationships of the S-Linked Glycocin Sublancin. ACS Chem. Biol..

[11] Ren H, Biswas S, Ho S, Van Der Donk WA, Zhao H (2018). Rapid Discovery of Glycocins through Pathway Refactoring in *Escherichia coli*. ACS Chem. Biol..

[12] Norris GE, Patchett ML (2016). The glycocins: in a class of their own. Curr Opin Struct Biol..

[13] Wang, S. *et al*. Prevention of Cyclophosphamide-Induced Immunosuppression in Mice with the Antimicrobial Peptide Sublancin. **2018**, 4353580 (2018). 10.1155/2018/4353580 PMC5964538 29854837

[14] Wang S (2017). Use of the antimicrobial peptide sublancin with combined antibacterial and immunomodulatory activities to protect against methicillin-resistant *Staphylococcus aureus* infection in mice. J. Agric. Food Chem..

[15] Garcia De Gonzalo CV (2015). The phosphoenolpyruvate: Sugar phosphotransferase system is involved in sensitivity to the glucosylated bacteriocin sublancin. Antimicrob. Agents Chemother..

[16] Hemphill HE, Gage I, Zahler SA, Korman RZ (1980). Prophage-mediated production of a bacteriocinlike substance by SPβ lysogens of *Bacillus subtilis*. Can. J. Microbiol..

[17] Lazarevic V (1999). Nucleotide sequence of the *Bacillus subtilis* temperate bacteriophage SPβc2. Microbiology.

[18] Paik SH, Chakicherla A, Norman Hansen J (1998). Identification and characterization of the structural and transporter genes for, and the chemical and biological properties of, sublancin 168, a novel lantibiotic produced by *Bacillus subtilis* 168. J. Biol. Chem..

[19] Kouwen TRHM (2007). Thiol-disulphide oxidoreductase modules in the low-GC Gram-positive bacteria. Mol. Microbiol..

[20] Dubois JYF (2009). Immunity to the bacteriocin sublancin 168 is determined by the SunI (YolF) protein of *Bacillus subtilis*. Antimicrob. Agents Chemother..

[21] Luo Y, Helmann JD (2009). Extracytoplasmic function?? factors with overlapping promoter specificity regulate sublancin production in *Bacillus subtilis*. J. Bacteriol..

[22] Strauch MA (2007). Abh and AbrB control of *Bacillus subtilis* antimicrobial gene expression. J. Bacteriol..

[23] Albano M (2005). The Rok protein of *Bacillus subtilis* represses genes for cell surface and extracellular functions. J. Bacteriol..

[24] Smits WK, Grossman AD (2010). The transcriptional regulator Rok binds A+T-rich DNA and is involved in repression of a mobile genetic element in *Bacillus subtilis*. PLoS Genet..

[25] Serizawa M (2005). Functional Analysis of the YvrGHb Two-Component System of *Bacillus subtilis* : Identification of the Regulated Genes by DNA Microarray and Northern Blot Analyses. Biosci. Biotechnol. Biochem..

[26] Nakano S, Küster-Schöck E, Grossman AD, Zuber P (2003). Spx-dependent global transcriptional control is induced by thiol-specific oxidative stress in *Bacillus subtilis*. Proc. Natl. Acad. Sci..

[27] Rochat T (2012). Genome-wide identification of genes directly regulated by the pleiotropic transcription factor Spx in *Bacillus subtilis*. Nucleic Acids Res..

[28] Lorca GL (2005). Catabolite repression and activation in *Bacillus subtilis*: Dependency on CcpA, HPr, and HprK. J. Bacteriol..

[29] Ogura M, Yamaguchi H, Yoshida K, Fujita Y, Tanaka T (2001). DNA microarray analysis of *Bacillus subtilis* DegU, ComA and PhoP regulons: an approach to comprehensive analysis of *B*. *subtilis* two- component regulatory systems. Nucleic Acids Res..

[30] Veening JW (2008). Transient heterogeneity in extracellular protease production by *Bacillus subtilis*. Mol. Syst. Biol..

[31] Bron S, Venema G (1972). Ultraviolet inactivation and excision-repair in *Bacillus subtilis* I. Construction and characterization of a transformable eightfold auxotrophic strain and two ultraviolet-sensitive derivatives. Mutat. Res. - Fundam. Mol. Mech. Mutagen..

[32] Tanaka K (2013). Building the repertoire of dispensable chromosome regions in *Bacillus subtilis* entails major refinement of cognate large-scale metabolic model. Nucleic Acids Res..

[33] Botella E (2010). pBaSysBioII: An integrative plasmid generating gfp transcriptional fusions for high-throughput analysis of gene expression in *Bacillus subtilis*. Microbiology.

[34] Kouwen TRHM (2009). The large mechanosensitive channel MscL determines bacterial susceptibility to the bacteriocin sublancin 168. Antimicrob. Agents Chemother..

[35] Schneider CA, Rasband WS, Eliceiri KW (2012). NIH Image to ImageJ: 25 years of image analysis. Nature Methods.

[36] Piersma S (2013). TLM-Quant: An Open-Source Pipeline for Visualization and Quantification of Gene Expression Heterogeneity in Growing Microbial Cells. PLoS One.

[37] Ireton K, Rudner DZ, Siranosian KJ, Grossman AD (1993). Integration of multiple developmental signals in *Bacillus subtilis* through the Spo0A transcription factor. Genes Dev..

[38] Chumsakul O (2011). Genome-wide binding profiles of the *Bacillus subtilis* transition state regulator AbrB and its homolog Abh reveals their interactive role in transcriptional regulation. Nucleic Acids Res..

[39] Banse AV, Chastanet A, Rahn-Lee L, Hobbs EC, Losick R (2008). Parallel pathways of repression and antirepression governing the transition to stationary phase in *Bacillus subtilis*. Proc. Natl. Acad. Sci..

[40] Hoa TT, Tortosa P, Albano M, Dubnau D (2002). Rok (YkuW) regulates genetic competence in *Bacillus subtilis* by directly repressing comK. Mol. Microbiol..

[41] De Jong IG, Veening JW, Kuipers OP (2010). Heterochronic phosphorelay gene expression as a source of heterogeneity in *Bacillus subtilis* spore formation. J. Bacteriol..

[42] Veening JW, Hamoen LW, Kuipers OP (2005). Phosphatases modulate the bistable sporulation gene expression pattern in *Bacillus subtilis*. Mol. Microbiol..

[43] Goelzer A (2015). Quantitative prediction of genome-wide resource allocation in bacteria. Metab. Eng..

[44] Kunst F (1997). The complete genome sequence of the gram-positive bacterium *Bacillus subtilis*. Nature.

[45] Boonstra M (2013). Spo0A regulates chromosome copy number during sporulation by directly binding to the origin of replication in *Bacillus subtilis*. Mol. Microbiol..

[46] Wiegert T, Schumann W (2001). SsrA-mediated tagging in *Bacillus subtilis*. J. Bacteriol..

[47] Blencke HM (2006). Regulation of *citB* expression in *Bacillus subtilis*: Integration of multiple metabolic signals in the citrate pool and by the general nitrogen regulatory system. Arch. Microbiol..

[48] Kobayashi K (2003). Essential *Bacillus subtilis* genes. Proc. Natl. Acad. Sci..

[49] Ludwig H, Rebhan N, Blencke HM, Merzbacher M, Stülke J (2002). Control of the glycolytic gapA operon by the catabolite control protein A in *Bacillus subtilis*: A novel mechanism of CcpA-mediated regulation. Mol. Microbiol..

[50] Jordan S (2007). LiaRS-dependent gene expression is embedded in transition state regulation in *Bacillus subtilis*. Microbiology.

[51] Guillen N, Weinrauch Y, Dubnau DA (1989). Cloning and characterization of the regulatory *Bacillus subtilis* competence genes *comA* and *comB*. J. Bacteriol..

