# Supplementary material for: Differential expression of a prophage-encoded glycocin and its immunity protein suggests a mutualistic strategy of a phage and its host
Source: Sci Rep. 2019 Feb 26;9:2845. doi: 10.1038/s41598-019-39169-3 (PMC6391423; doi:10.1038/s41598-019-39169-3)
Supplement: Supplementary file 1 — Supplementary Figures S1, S2 and S3 [file 41598_2019_39169_MOESM1_ESM.pdf]

Supplementary information

Differential expression of a prophage-encoded glycocin and its immunity protein suggests a mutualistic strategy of a phage and its host

Emma L. Denham[1,2#], Sjouke Piersma[1#], Marleen Rinket[1], Ewoud Reilman[1], Marcus C. de Goffau[1,3], and Jan Maarten van Dijl*[1]

[1]University of Groningen, University Medical Center Groningen, Department of Medical Microbiology, Hanzeplein 1, P.O. Box 30001, 9700 RB Groningen, the Netherlands

[2]Present address: Department of Biology and Biochemistry, University of Bath, Bath, UK

[3]Present address: Wellcome Sanger Institute, Cambridge, UK

[#]These authors contributed equally to this work

***Corresponding author:** Jan Maarten van Dijl, Department of Medical Microbiology, University of Groningen, University Medical Center Groningen, Hanzeplein 1, P.O. Box 30001, 9700 RB Groningen, the Netherlands, Tel. +31-50-3615187, Fax. +31-50-3619105, E-mail: j.m.van.dijl01@umcg.nl

Running title: Sublancin expression heterogeneity in *Bacillus*

Supplementary Figure S1

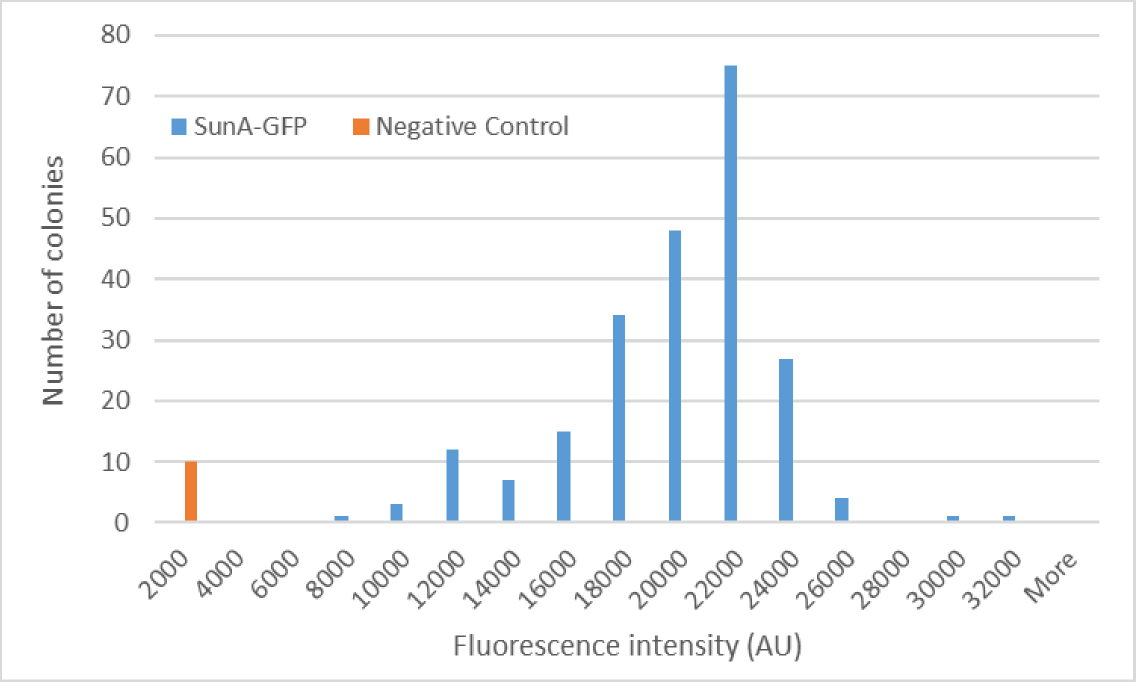

Supplementary Figure S1. Histogram of colony fluorescence intensity to verify the stable maintenance of the integrated P*sunA*-GFP construct.

B. subtilis strain P*sunA*-GFP was grown overnight in LB medium without antibiotics. The next day, a 100 ul aliquot of a 10000x dilution of the overnight culture was plated on LB-agar without antibiotics. Upon overnight incubation of the plates, the colonies were imaged for GFP fluorescence on an Amersham Typhoon imager, and the fluorescence intensities of all colonies on a plate were measured using ImageJ. From the intensities a histogram was created depicting the number of colonies with a particular fluorescence intensity in arbitrary units (AU). None of the colonies on a control plate with *B. subtilis* 168 showed fluorescence of > 2000 AU.

Supplementary Figure S2

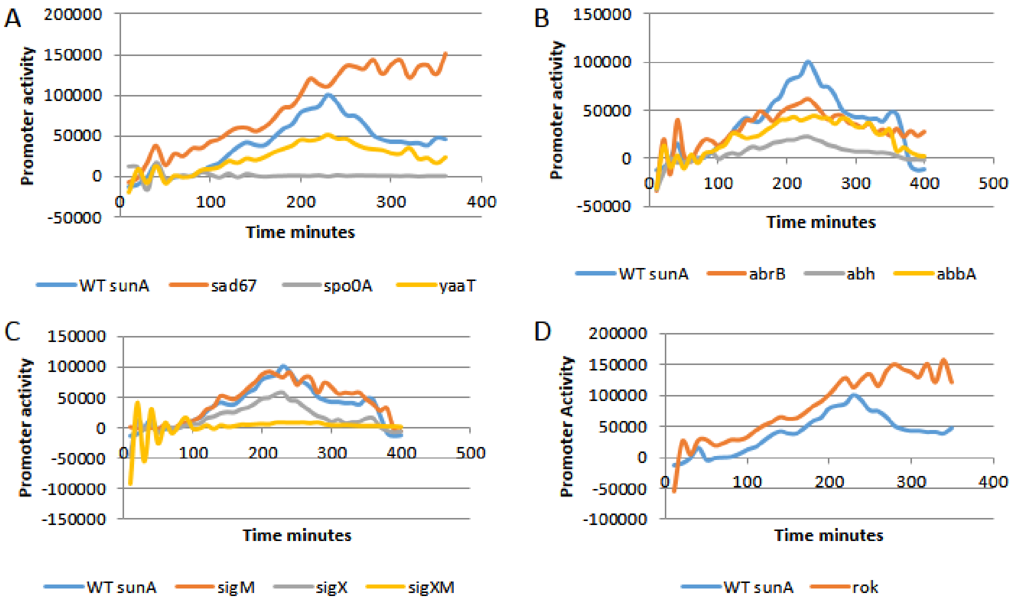

Supplementary Figure S2. The expression of *sunA* is influenced by various regulators.

B. subtilis cells with a *sunA* promoter GFP fusion were grown in microtiter plates and GFP fluorescence was recorded at 10 min intervals. Promoter activity was calculated and plotted for the parental strain 168 (blue line) and particular mutant strains. The different tested mutations are indicated in the legend below panels A-D.

Supplementary Figure S3

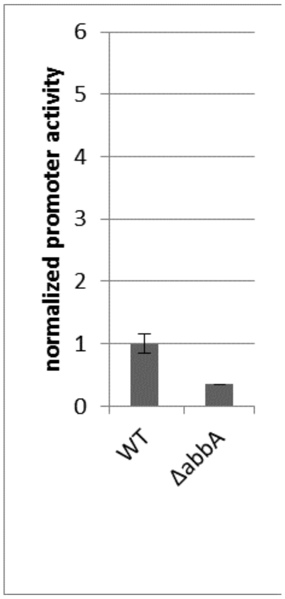

Figure S3. Maximum *sunA* promoter activity in different *B. subtilis* mutant strains.

The maximum *sunA* promoter activity value for the investigated *abbA* mutant strain was determined by time-lapse fluorescence microscopy as in Figure 2, and normalized against the maximum promoter activity determined for the parental strain 168 (WT).
